# The lipoxygenase OsLOX10 affects seed longevity and resistance to saline-alkaline stress during rice seedlings

**DOI:** 10.1007/s11103-023-01334-8

**Published:** 2023-03-03

**Authors:** Fuxiang Wang, Huibin Xu, Ling Zhang, Yunrui Shi, Yu Song, Xinyue Wang, Qiuhua Cai, Wei He, Huaan Xie, Jianfu Zhang

**Affiliations:** 1 College of Agriculture, Fujian Agriculture and Forestry University, 350002 Fuzhou, China; 2 Rice Research Institute, Fujian Academy of Agricultural Sciences, 350018 Fuzhou, China; 3 State Key Laboratory of Ecological Pest Control for Fujian and Taiwan Crops/Key Laboratory of Germplasm Innovation and Molecular Breeding of Hybrid Rice for South China, Incubator of National Key Laboratory of Germplasm Innovation and Molecular Breeding between Fujian and Ministry of Sciences and Technology/Fuzhou Branch, National Rice Improvement Center of China/Fujian Engineering Laboratory of Crop Molecular Breeding/Fujian Key Laboratory of Rice Molecular Breeding, Ministry of Agriculture and Affairs, 350003 Fuzhou, China

**Keywords:** Lipoxygenase OsLOX10, Seed longevity, Free fatty acid, Saline-alkaline stress

## Abstract

Prolonged storage of rice seeds can lead to a decrease in seed vigor and seedling quality. The Lipoxygenase (LOX) gene family is widely distributed in plants, and LOX activity is closely related to seed viability and stress tolerance. In this study, the lipoxygenase *OsLOX10* gene from the 9-lipoxygenase metabolic pathway was cloned from rice, and its roles in determining seed longevity and tolerance to saline-alkaline stress caused by Na_2_CO_3_ in rice seedlings were mainly investigated. CRISPR/Cas9 knockout of *OsLOX10* increased seed longevity compared with the wild-type and *OsLOX10* overexpression lines in response to artificial aging. The expression levels of other 9-lipoxygenase metabolic pathway related genes, such as *LOX1*, *LOX2* and *LOX3*, were increased in the *LOX10* overexpression lines. Quantitative real-time PCR and histochemical staining analysis showed that the expression of *LOX10* was highest in seed hulls, anthers and the early germinating seeds. KI-I_2_ staining of starch showed that LOX10 could catalyze the degradation of linoleic acid. Furthermore, we found that the transgenic lines overexpressing *LOX10* showed better tolerance to saline-alkaline stress than the wild-type and knockout mutant lines. Overall, our study demonstrated that the knockout *LOX10* mutant increased seed longevity, whereas overexpression of *LOX10* enhanced tolerance to saline-alkaline stress in rice seedlings.

**Supplementary Information:**

The online version contains supplementary material available at 10.1007/s11103-023-01334-8.

## Key message

The Lipoxygenase *OsLOX10* gene was cloned from rice (*Oryza sativa* L.). Mutation in *LOX10* caused increasing of seed longevity, whereas overexpression of *LOX10* enhanced tolerance to saline-alkaline stress in rice seedlings.

**Supplementary Information:**

The online version contains supplementary material available at 10.1007/s11103-023-01334-8.

## Introduction

Plant processes are often affected by environmental stresses, including drought, high and low temperature, saline-alkaline stress, wounding, etc., which have a negative effect on the growth and development of the plant as a result of inhibition of metabolism and changing gene expression patterns. The lipoxygenase pathway (LOX-pathway) metabolites polyunsaturated fatty acids (PUFAs), participate in plant adaptation to stress by sensing and responding to signals, enhancing the expression of defense genes (Feussner and Wasternack 2002; Porta and Rocha-Sosa 2002). The signal molecules generated in plants by the LOXs-pathway are involved in a variety of functions, including growth and development, seed germination, response to biotic and abiotic stresses, wounding, fruit ripening, senescence, cell death, and the biosynthesis of the stress-response phytohormones jasmonic acid and abscisic acid (Viswanath et al. 2020). Previous studies on LOXs have focused mainly on their functions in development and in defense responses to biotic stress (Marla and Singh 2012), where studies showed increases in *LOX* gene expression and in the corresponding enzyme activity in response to pests and fungal phytopathogens such as blast in rice (Peng et al. 1994). Oxygen free radicals and hydroxide formed by the LOX pathway act as signal transduction substances to participate in plant responses to biotic stresses, but comparatively few studies have been carried out to establish the role of LOXs in plant response to abiotic stress. Studies have shown that the LOX pathway of membrane lipid conversion is an independent signaling pathway (Santino et al. 2013). This signal is enhanced by an autocatalytic cycle involving calcium ions and the calcium-binding protein calmodulin. Hydroperoxides produced by LOX activity on linoleic acid and linoleic acid in plasma membrane transport calcium ions from the outside of cells to the inside of cells (Feussner and Wasternack 2002; Glyan’ko, 2011). The subsequent increase in cytoplasmic calcium concentration leads to the activation of phospholipase A and the release of PUFAs from phospholipids (Babenko et al. 2017; Liavonchanka and Feussner 2006). LOX plays an important role as a key cascade enzyme in this process. Moreover, the intermediate and end products of the LOX pathway can activate protein kinases, sending signals and achieve their transduction. As a consequence, LOX activity is regarded as a molecular marker for plant response to abiotic stresses (Babenko et al. 2017; Pokotylo et al. 2015).

Rice (*Oryza sativa* L.) is one of the most important crops worldwide, and its yield has been increasing in recent years. Rice seeds are faced with a series of problems in the process of seed production and storage, such as decay, decline in vigor and germination rate, as well as disimprovements in consumer traits such as viscosity, hardness, taste and flavor; these changes are closely related to the oxidative decomposition of membrane lipids in the seeds (Yasumatsu and Moritaka 1964; Yasumatsu et al. 1966). How to maintain seed longevity and prevent quality decline during long-term storage is a universal problem. Many factors affecting seed longevity and quality during storage, including environmental conditions, seed physiological status and sub-species (*japonica* vs. *indica*) differences (Vertucci and Roos 1990; Zhang et al. 2007). Lipid degradation is considered to be one of the reasons for the decline in seed longevity and quality during storage (Takano 1993). Lipids can be metabolized by different enzyme systems (LOXs, hydrolases or lyases) into small molecules of alcohols, aldehydes and other volatiles during seed storage, resulting in lower nutritional value (Aibara et al. 1986).

In plants, linolenic acid (LNA) and linoleic acid (LA) are the most common substrates for LOXs; LOXs generate 9- or 13-hydrogen peroxides by adding oxygen at the 9- (9-LOX) or 13- (13-LOX) positions of the substrate carbon chain, respectively (Ivanov et al. 2010; Liavonchanka and Feussner 2006). Inhibition of LOXs activity affects seed storage stability by delaying the metabolism of PUFAs in seeds, maintaining the structual integrity of cell membranes and reducing the formation of cytotoxic derivatives. To date, several *LOX* family genes have been isolated from rice (Umate 2011). Among them, *LOX2* and *LOX3* were isolated from rice embryos and their expression is closely related to seed germination and the duration of vigor longevity in response to storage (Huang et al. 2014; Xu et al. 2015), but little is known about the effects of other *LOXs* on seed longevity during storage.

Seed aging is a complex process, which involves physiological and biochemical reactions, signal transduction, metabolic activation, redox homeostasis, etc. (Parkhey et al. 2012). The metabolites produced by lipid peroxidation, such as malondialdehyde (MDA) and acetaldehyde, can react with some macromolecules to cause cell damage, which not only affects the longevity and quality of seeds, but is also closely involved in determining the damage caused to plants by stresses. In the current study, rice *OsLOX10* was cloned from three-day-old seedlings of rice cultivar Kitaake with the intention of investigating its function in seed longevity and tolerance of rice seedlings to saline-alkaline stress. Knockout of *LOX10* increased seed longevity after artificial aging for 18 days. Lines overexpressing *LOX10* showed accelerated seed germination under normal condition and lower seed viability after artificial aging for 18 days; in addition, lines overexpressing *LOX10* showed higher tolerance to saline-alkaline stress than the wild-type (WT) and the knockout mutant lines. The practical application of *LOX10* might contribute to achieving genetic improvement of rice seed longevity and increased tolerance to saline-alkaline stress.

## Materials and methods

### Plant materials

The *japonica* cultivars variety Kitaake was used as the WT material for *Agrobacterium tumefaciens*-mediated transformation to construct the *OsLOX-OE* lines and for the generation of the *lox10* knockout mutants achieved using the CRISPR/Cas9 system, from which two independent homozygous mutants *lox10-1* and *lox10-2* were identified using specific primers (Table S1). The overexpression vector of *LOX10* was constructed based on the pCAMBIA-1302 plasmid driven by a CaMV 35 S promoter. The transgenic plants of T_1_ generation were screened with primers of the hygromycin phosphotransferase gene and homozygous T_2_ generation transgenic seeds were obtained (Table S1). Plants of the lines were grown in an experimental field and harvested uniformly at the maturity stage, with all seeds subsequently undergoing the same storage conditions.

### Saline-alkaline stress treatment

To conduct the saline-alkaline stress treatment, rice seedlings were grown for three weeks at a 28℃ temperature, under a 16-h light/8-h dark photoperiod, with approximately 70% relative humidity and 500 µmol photons m^− 2^s^− 1^ light intensity. Saline-alkaline stress treatment was conducted as described in a previous reports (Liu et al. 2021). After saline-alkaline stress treatment for 48 h, the MDA concentration was determined by the MDA Assay Kit (Solarbio, Beijing, China), following the manufacturer’s instructions. Three biological replicates were performed for each treatment sample. A section of the flag leaf section from each mature plants was floated in a Petri dish on a solution containing 25 mM Na_2_CO_3_ ( pH = 10.0 ) and incubated for three days, after which the leaf was scored for necrosis or spots, and photographic images were taken. Chlorophyll a, chlorophyll b and total chlorophyll content of entire leaves from in WT, *lox10* mutants and *LOX10* overexpression lines were determined according to Zhang et al. (2015).

### Seed germination and artificial aging

Fifty seeds per replicate of each of the *lox10* mutants, the *LOX10* overexpressed lines and WT ‘Kitaake’ were placed in 9-cm diameter Petri dishes containing 10 ml of distilled water in an incubator at 28 ± 1 °C, 100% relative humidity and 16 h of light per day for seven days. Seed vigor traits were measured according to previous reports, namely germination potential, germination rate and germination index (Fu et al. 2019; He et al. 2019). Three biological replicates were performed for each sample. The artificial aging treatment procedure was based on the method of Zeng et al. (Zeng et al., 2007) with some modifications. Fifty seeds per replicate of the *lox10* mutants, the *LOX10* overexpression lines and WT were treated at 42 °C and 88% relative humidity for 18 days in a closed desiccator (Binder, Tuttlingen, Germany) with a thermostatic moisture regulator. After artificial aging, the germination potential at 7 days and the germination rate at 14 days were measured according to the above method.

### RNA isolation and gene expression analysis

Total RNA was isolated from different tissues (roots, stems, leaves, embryos) of rice seedlings, germinated seeds (after 0, 1, 3, 5 and 7 days of incubation) and transgenic embryos using the TRIzol reagent (Simms et al. 1993). The first-strand cDNA was synthesized from 1 µg of total RNA using ReverTra Ace™ qPCR RT Master Mix with gDNA Remover (TOYOBO, Osaka, Japan). Real-time quantitative PCR was performed using the LightCycler 480 II ( Roche, Jena, Germany) and the FastStart Universal SYBR Green Master (Rox) (Roche, Shanghai, China), with the rice *OsActin* gene as the internal control. The PCR conditions were as follows: 95℃ for 10 min, followed by 40 cycles at 95℃ for 15 s and 60℃ for 60s. Primers used for qPCR are listed in Supplemental Table S1. Data were analyzed using LightCycler 480 II software.

### Cloning of *OsLOX10*

Total RNA was isolated from three-day-old seedlings of ‘Kitaake’ to carry out *OsLOX10* cloning. Based on the *OsLOX10* mRNA sequence XM_015759992 in the NCBI database (https://www.ncbi.nlm.nih.gov/), we designed primer pairs LOX10-CDS-F and LOX10-CDS-R (Supplemental Table S1), which amplified the *LOX10* coding sequence (CDS) (2607 bp) . Meanwhile, we designed primers LOX10-Pro-F and LOX10-Pro-R (Supplemental Table S1) to amplify the 2000 bp sequence upstream of *LOX10*-CDS as the promoter of *LOX10* gene from the ‘Kitaake’ genome.

### GUS staining

The 2-kb promoter region of *LOX10* was amplified with the primer sequences listed in Supplemental Table S1. Cloning of the sequence into *BamHI* and *NcoI* sites of the pCAMBIA1305 plasmid was conducted using the *BamHI* and *NcoI* restriction enzymes. The vector was introduced into ‘Kitaake’ by *A.tumefaciens-mediated transformation*. GUS staining of different tissues of transgenic plants was conducted overnight using a commercial GUS Staining Solution Kit (Beijing, Solarbio, China) at 37℃ according to the manufacturer’s instructions. Photographic images of the tissues were obtained after decolorization of the tissues with 75% (v/v) alcohol.

### Protein subcellular localization

The CDS of *LOX10* was amplified with the primer sequences listed in Supplemental Table S1. The pRTVcGFP plasmid was digested with *BamHI* and *HindIII* restriction enzymes to construct the *LOX10*::*GFP* vector. Rice protoplasts were prepared and transfected as described in a previous report (Zhang et al. 2011). Scanning observation of fluorescent proteins were carried out under a TCS SP8 STED 3X laser confocal microscope ( Leica, Germany).

### *In-vitro* expression and enzyme activity assays

The CDS of *LOX10* was inserted into the pET28a plasmid and double digested with *NdeI* and *HindIII* to construct the pET28a(+)::*LOX10* vector. Then, the pET28a::*LOX10* plasmid and the pET28a control plasmid were transformed separately into cells of the *Escherichia coli* BL21(DE3) prokaryotic expression strain and expression of the fusion protein product was induced by exposure to 0.1 mM isopropylthio-β-D-galactoside (IPTG) for 5 h at 23℃. The *E.coli* cells were collected, exposed to ultrasonic treatment in phosphate-buffered saline (PBS) on ice and then centrifuged. LOX10 activity was assayed in the supernatant and in transgenic seed embryos using the KI-I_2_ starch staining method (Xu et al. 2015). The stock LOX10 supernatant, which contained 0.5 µg protein/µl, was diluted to achieve different working concentrations (1×, 2×, 5×dilutions) for the enzyme activity assay and the pET28a(+) supernatant and PBS buffer were used as controls. The color intensity of the different reaction solutions reflected the activity level of LOX10.

### Measurement of free fatty acid concentration

The transgenic and WT seeds were artificially aged for 18 days, as described earlier, and approximately 0.5 mg of embryos were then dissected from the seeds, snap frozen in liquid nitrogen and sent to Biotree (Shanghai, China) for determination of free fatty acid concentrations. The embryos from transgenic and WT seeds stored under natural conditions were used as controls. Three biological replicates were performed for each treatment.

### SEM analysis

Transgenic and WT seeds before and after artificially aging for 18 days were viewed under a scanning electron microscope (SEM) (Xu et al. 2015). Rice seed endosperm was cut transversely and placed in a S-3500 N SEM (Hitachi, Tokyo, Japan ) and images were recorded according to the manufacturer’s protocol.

### Agronomic characteristics of transgenic rice lines and data analysis

The grain length, grain width and 1000-grain weight of transgenic and WT plants were determined. Three biological replicates were performed for each line. Student’s *t*-test was used to determine whether any pairwise differences between samples were statistically significant, with significance being assessed at the 0.05 and 0.01 levels.

## Results

### Disruption of *LOX10* expression increase seed longevity

**Fig. 1:**
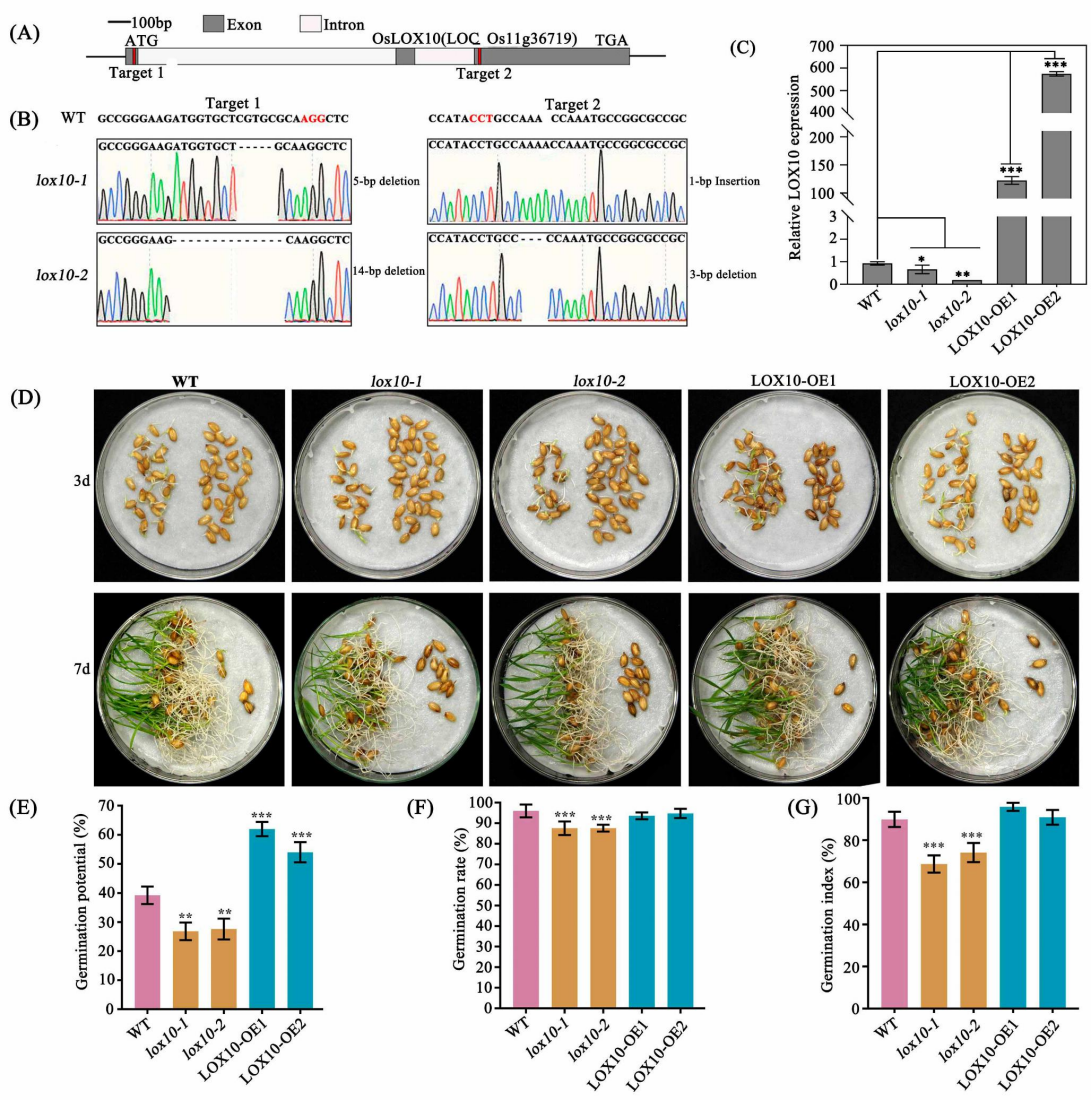
**The mutation of*LOX10*affects rice seed vigor**. (a) Gene structures of WT and *lox10* mutants,. The red box represents the CRISPR/Cas9 target of the *LOX10* gene. (b) The *lox10-1* mutant plants contained a -5 bp deletion and a + 1 bp insertion, the *lox10-2* mutant plants contained a -14 bp and a -3 bp deletion. (c) Relative expression analysis of the *LOX10* gene in WT, *lox10* knockout mutants and *LOX10* overexpression lines. (d) Seed germination of WT, *lox10* mutants and *LOX10* overexpression lines after 3 and 7 days under normal conditions. (e-g) Germination potential, germination rate and germination index of WT, *lox10* mutants and *LOX10* overexpression lines after 7 days under normal conditions. Values are means ± SD of three biological replicates; *, ** and *** indicate the significant difference compared to WT at 5%, 1% and 0.1% levels, respectively, according to Student’s t-test

Rice *LOX10*, which is located on chromosome 11 and encodes a protein of 868 amino acids (Fig. S1a,b), was cloned from a *japonica* variety, Kitaake. *LOX10* belongs to the 9-LOX metabolic pathway according to KEGG (https://www.genome.jp/kegg/) database analysis. The phylogenetic tree indicated that a closer genetic relationship of *OsLOX10* exists among the monocotyledonous plants rice, wheat, and maize than among the dicotyledonous plants *Arabidopsis*, soybean, and tomato (Fig. S1c). To extend our understanding of *LOX10 *functions, we generated knockout mutants (*lox10-1* and *lox10-2*) and overexpression lines (*LOX10-OE1* and *LOX10-OE2*) (Fig. 1a-c). The *lox10-1* mutant plants contained a -5 bp deletion and a + 1 bp insertion, whereas the *lox10-2* mutant plants contained a -14 bp deletion and a -3 bp deletion (Fig. 1b). In the two knockout mutants, the amino acid sequence of LOX10 suffered premature termination as a result of frame shift mutation of the nucleotide sequence (Fig. S2). These results indicate that the *lox10-1* and *lox10-2* mutant lines lacked LOX10 activity. In the *LOX10-OE1* and *LOX10-OE2* overexpression lines, the expression of *LOX10* was increased 122- and 574-fold, respectively, compared with the WT (Fig. 1c). The self progeny of these homozygous mutants were used in subsequent experiments.

Seed vigor is an important factor affecting seed germination and longevity. Our data showed that there were no significant differences for agronomic traits, including plant architecture, grain length, grain width and 1000-grain weight among the WT, the mutant and transgenic lines(Fig. S3a-f). The seeds of the two *lox10* mutants, the two overexpression lines and the WT were compared for the evaluation of seed vigor. Phenotype evaluation indicated that the disruption of *LOX10* in the two knockout mutants resulted in decreased seed vigor reflected by slow germination speed and slow seedling growth under normal conditions (Fig.S3g-i). The germination potential, germination rate and seedling vigor index of the two *lox10* mutants were significantly lower than the corresponding values of the two *LOX10* overexpression lines and the WT plants (Fig. 1d-g), whereas the germination potential of the two *LOX10* overexpression lines was significantly greater than that of the WT under normal conditions (Fig. 1e).

**Fig. 2:**
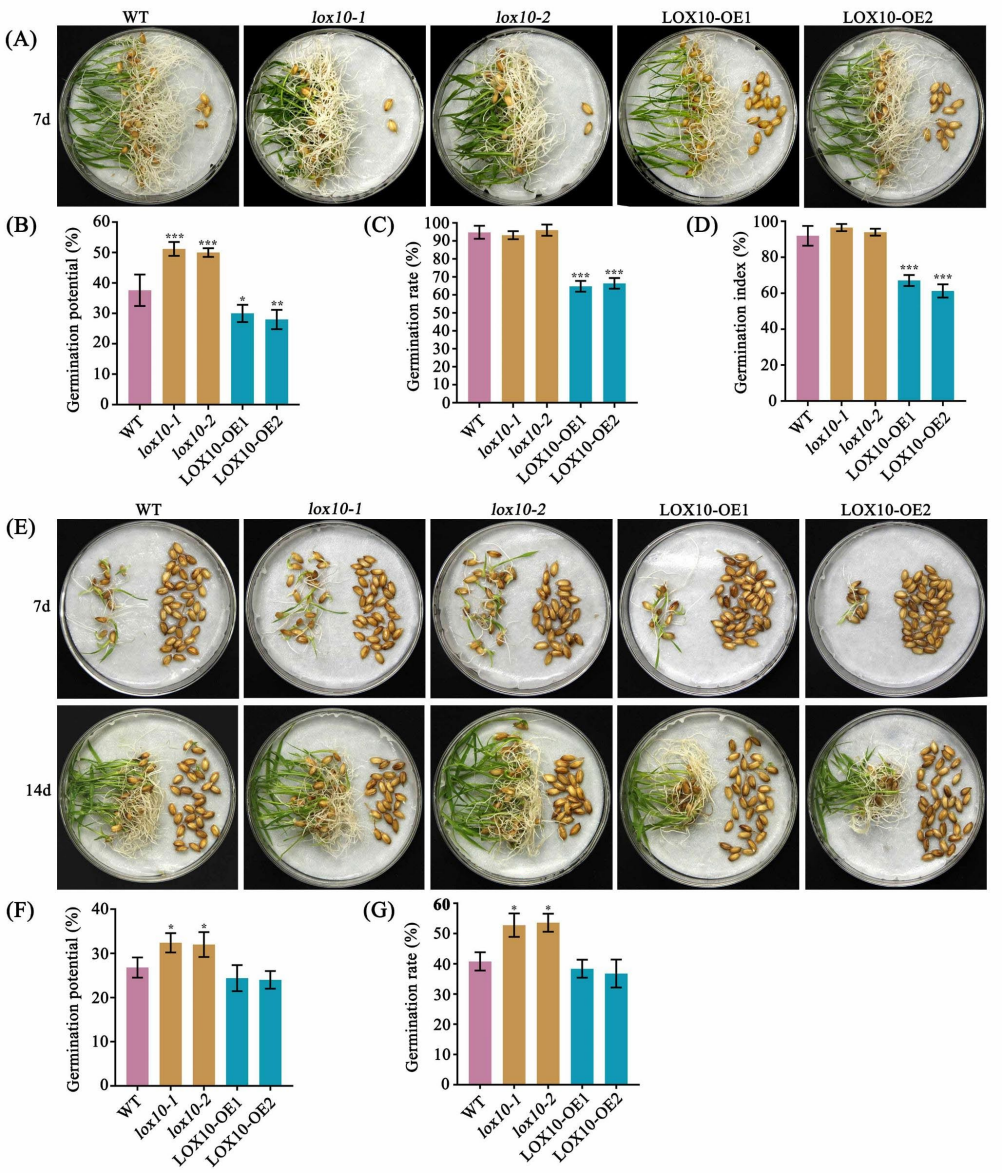
**Comparison of rice seed vigor among WT**, ***lox10*knockout mutants and*LOX10*overexpression lines under naturally aged and artificially aged conditions.** (a) Seed germination of WT, *lox10* mutants and *LOX10* overexpression lines after 7 days following natural aging for 6 months; (b-d) Germination potential, germination rate and germination index of WT, *lox10* mutants and *LOX10* overexpression lines after 7 days following natural aging for 6 months; (e) Seed germination of WT, *lox10* mutants and *LOX10* overexpression lines after 7 and 14 days following artificial aging for 18 days; (f-g) Germination potential and germination rate of WT, *lox10* mutants and *LOX10* overexpression lines after 14 days following artificial aging for 18 days. Values are means ± SD of three biological replicates; *, ** and *** indicate the significant difference compared to WT at 5%, 1% and 0.1% levels, respectively, according to Student’s t-test

Seed longevity was measured as the germination potential, germination rate and germination index after artificial aging (high temperature and high humidity treatment to accelerate seed aging) or natural aging treatments. After storage for 6 months under natural conditions, the germination potential of two *lox10* mutants was significantly higher than the WT, while the two *LOX10* overexpression lines were significantly lower than the WT (Fig. 2a,b). The germination rate and germination index of the two *lox10* mutants and WT were all remain above 90%, while the two *LOX10* overexpression lines were significantly reduced (Fig. 2a,c,d). Overall, the transgenic lines and WT exhibited similar trends with little difference under both natural and artificial aging conditions (Fig. 2). The germination potential and germination rate of the two *lox10* mutants were significantly higher than those of the two *LOX10* overexpression lines and WT plants after artificial aging for 18 days (Fig. 2e-g). All these results suggest that *LOX10* plays an important role in regulating seed vigor and longevity.

### Expression pattern of *LOX10* and subcellular localization

**Fig. 3:**
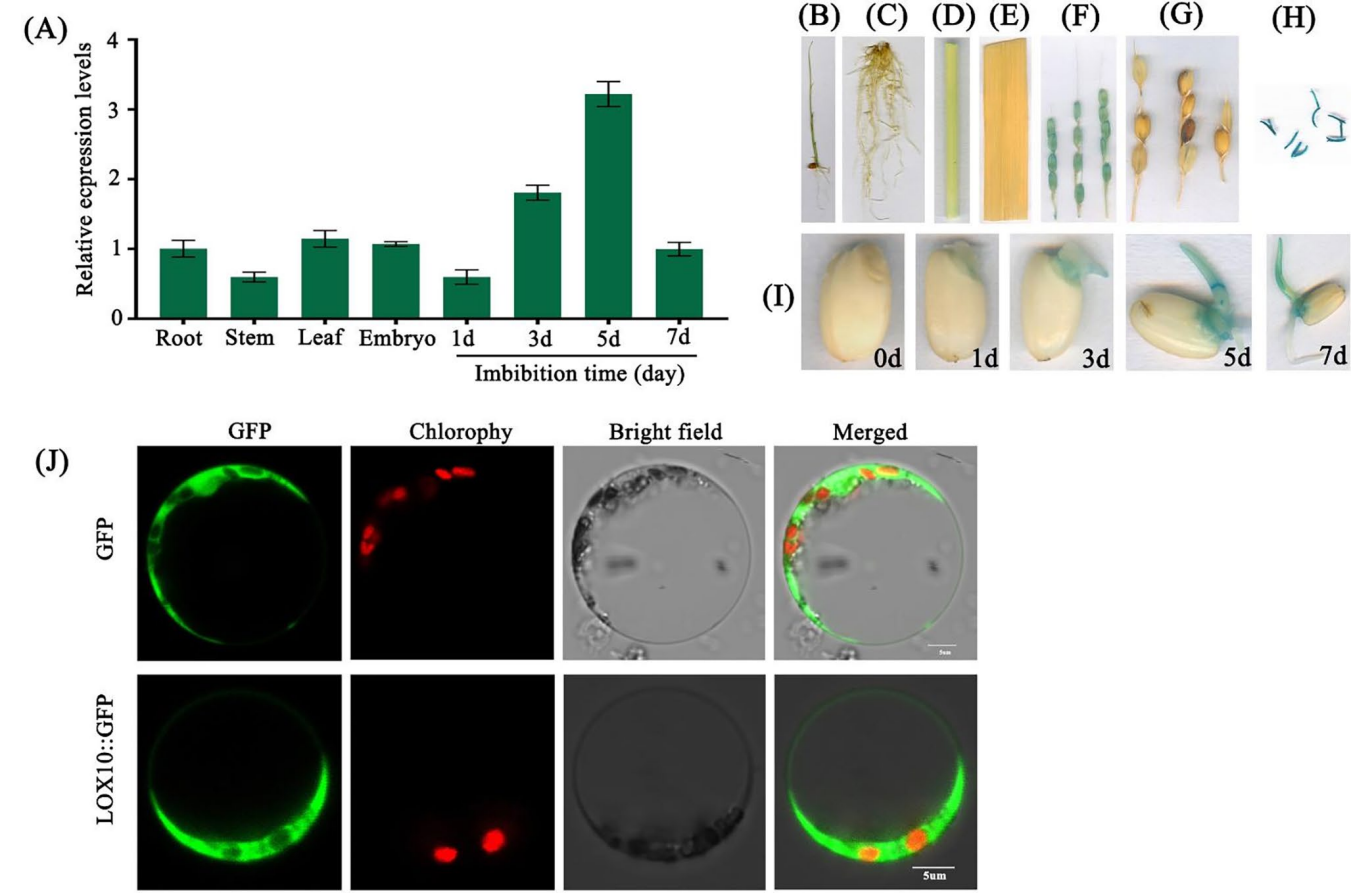
**Expression patterns of*LOX10*and subcellular localization in rice**. (a) Expression patterns of *LOX10* in different tissues and germination stages of in rice using the quantitative real-time PCR (qPCR) approach; (b-i)Histochemical staining for GUS activity of *LOX10* in seedling, root, stem, leaf, young panicle, mature panicles, anther and early seed germination stage; (j) Subcellular localization in rice protoplasts of LOX10 tagged with green fluorescent protein (GFP) at the C-terminus. Bar = 5 μm

To further understand the physiological function of *LOX10* in rice, the expression patterns of *LOX10* during seed germination and seedling development were analyzed using quantitative real-time PCR (qPCR). *LOX10* transcripts were detected during the early stage of seed germination (Fig. 3a). Histochemical staining for GUS activity was performed in *LOX10* promoter::GUS transgenic lines, and observed that GUS activity was relatively high in hull tissue, anther tissue and during the early seed germination stage (Fig. 3b-i). The elevated expression of *LOX10* in germinated seeds suggests that this gene might play an important role in modulating seed vigor in rice. We then further constructed a recombinant *LOX10* tagged with *GFP* at the C-terminus and expressed it transiently in rice protoplasts to determine the subcellular localization of *LOX10*. We observed the green fluorescence of GFP-tagged LOX10 to be distributed throughout the cytoplasm (Fig. 3j), indicating that LOX10 protein is present in the cytoplasm.

### LOX10 activity assay on α-linoleic acid (LA)

**Fig. 4:**
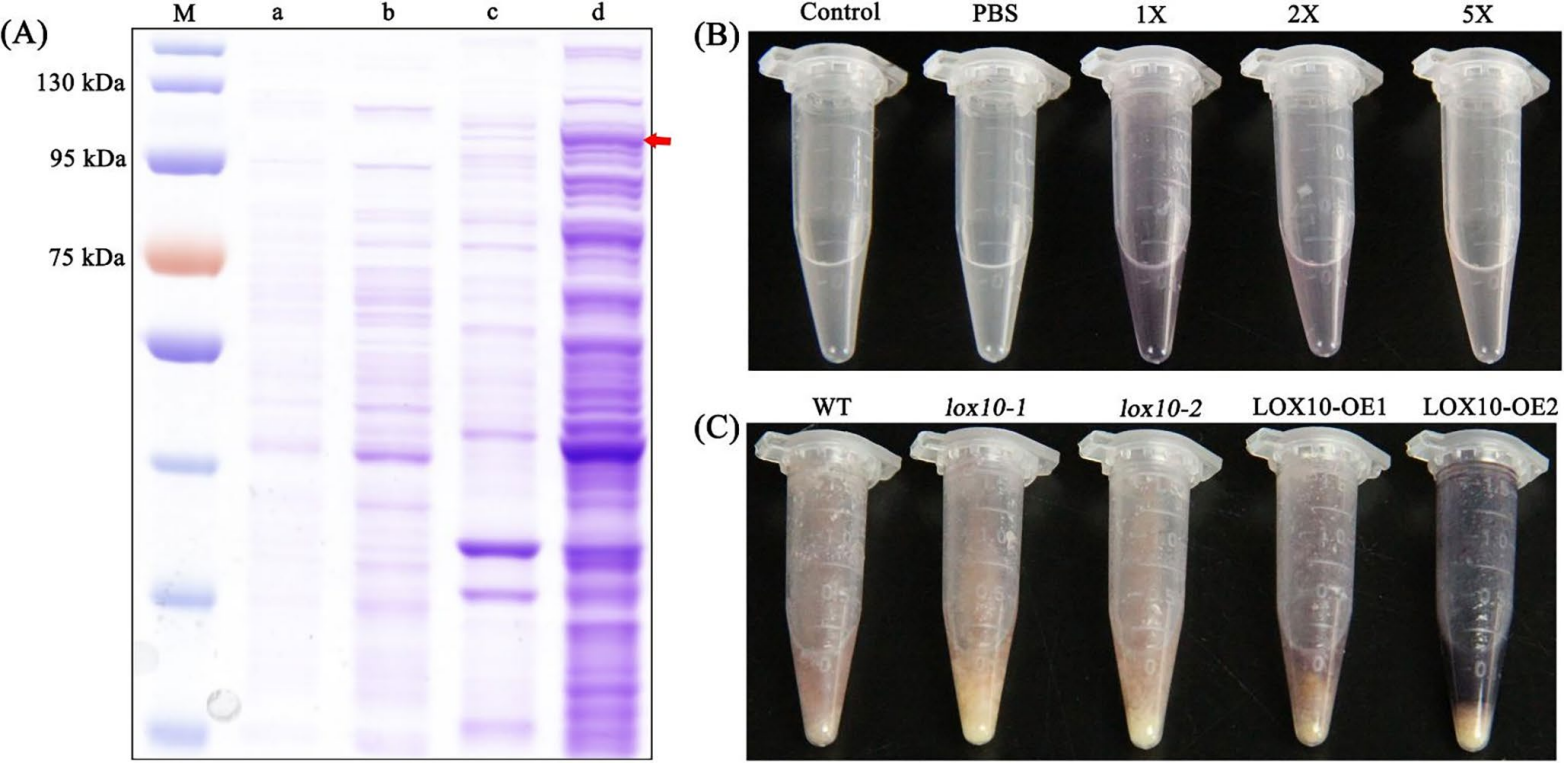
**Induction of LOX10 protein*in vitro*and determination of enzyme activity using the KI-I**_**2**_**starch staining method**. (a) Induction of production of LOX10 protein in vitro; m:protein marker; a: pET28a(+) precipitate; b: pET28a(+) supernatant; c: pET28a(+)::*LOX10* precipitate; d: pET28a(+)::*LOX10* supernatant; red arrow represents LOX10 protein. (b) Enzymatic activity assay of LOX10 on α-linoleic acid (LA) in vitro; control:pET28a(+) supernatant; PBS: PBS buffer; 1×dilution: the 0.5 µg protein/µl supernatant containing LOX10; 2×dilution: the 0.25 µg protein/µl supernatant containing LOX10; 5×dilution: the 0.1 µg protein/µl supernatant containing LOX10. (c) Enzyme activity assay of LOX10 on LA in WT, *lox10* mutants and *LOX10* overexpression rice seed embryos

We introduced complete LOX10 cDNA into pET28a(+) for *in-vitro* expression analysis. Using SDS-PAGE and western blotting, a specific band corresponding to 100 kDa was observed, consisting of LOX10 ( about 98 kDa ) and a His tag (Fig. 4a). Most of LOX10 recombinant proteins were present in the supernatant of the cell lysate under moderate-temperature induction conditions (23℃) and the recombinant protein exhibited high LOX10 activity (Fig. 4a). The supernatant was shown to contain the soluble LOX10-His fusion protein ( diluted to achieve different concentrations ) and the His tag protein were obtained in the supernatant for enzyme activity analysis. The KI-I_2_ staining showed that LOX10 could metabolize the substrate LA to produce starch, which stained dark purple, with the color becoming lighter (reflecting lower enzyme activity) as the protein concentration decreased (Fig. 4b). We separately extracted the embryo proteins from the lines *lox10-1*, *lox10-2*, *LOX10-OE1*, *LOX10-OE2* and WT for KI-I_2_ staining, and found that the two *LOX10-OE* overexpression lines showed deeper purple (higher activity), compared with the two *lox10* mutants and the WT (Fig. 4c). We speculated that the cause of the light purple staining by the *lox10* mutants and WT may be functional redundancy among LOX family members. These results showed that *LOX10* encoded an authentic LOX protein, which exhibited enzymatic activity.

### Disruption of *LOX10* affects grain starch granule structure and free fatty acid content

**Fig. 5:**
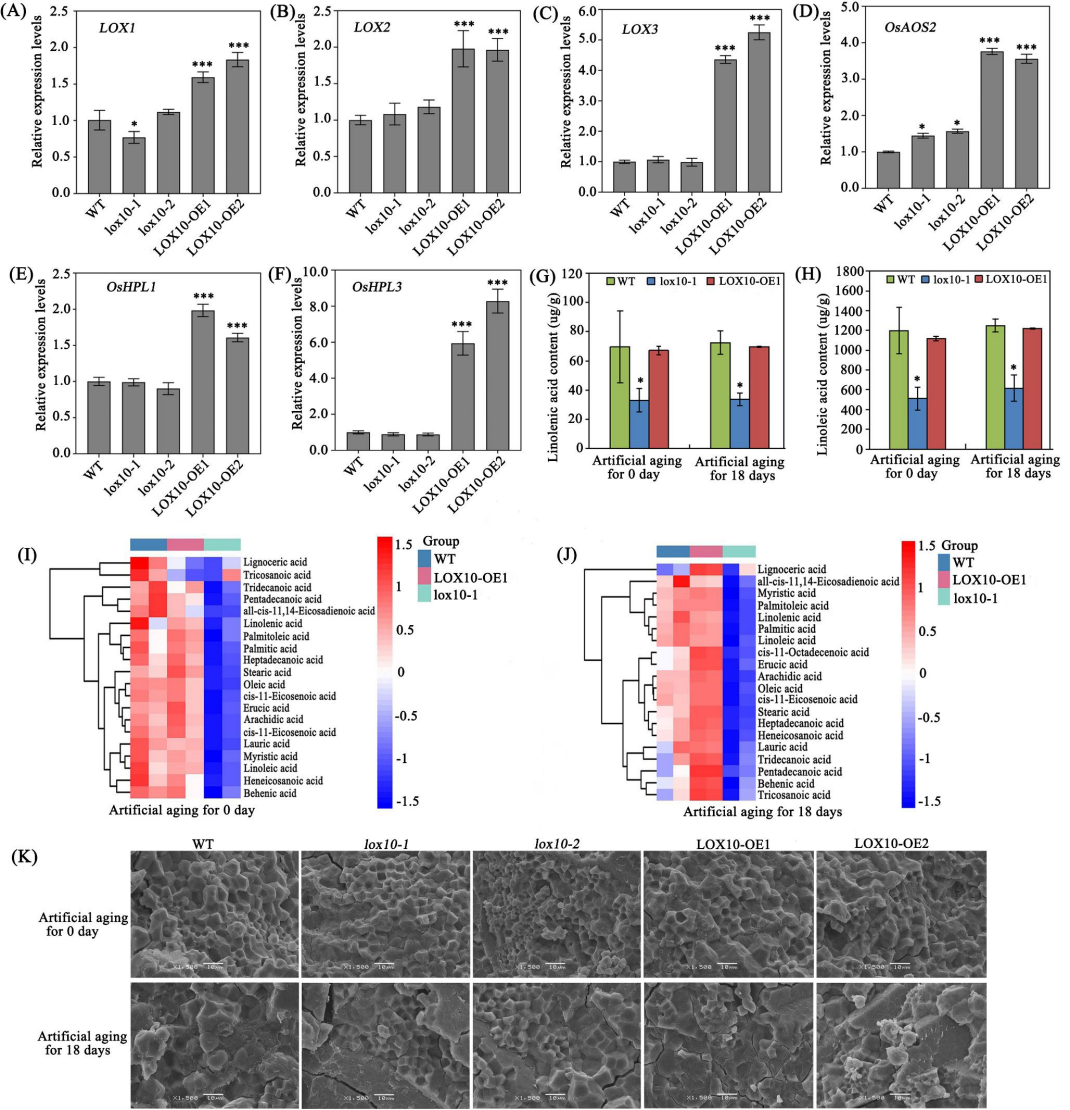
**Observation of starch granule structure in endosperm and determination of free fatty acid concentration in embryo**. (b) (a-c) Expression analysis of 9-LOX metabolic pathway-related genes *LOX1*, *LOX2* and *LOX3* in WT, *lox10* mutants and *LOX10* overexpression rice seed embryos. (d-f) Expression analysis of 9-LOX downstream metabolism-related genes *AOS2*, *HPL1* and *HPL3* in WT, *lox10* mutants and *LOX10* overexpression rice seed embryos. (g, h) Linolenic acid and linoleic acid concentrations in rice embryos of WT, *lox10-1* mutant and *LOX10-OE1* overexpression lines with or without artificial aging for 18 days. Values are means ± SD of three biological replicates; * and *** indicate the significant difference compared to WT at 5% and 0.1% levels, respectively, according to Student’s t-test. (i, j) Heatmap of metabolite clustering in rice embryos of WT, *lox10-1* mutant and *LOX10-OE1* overexpression lines with or without artificial aging for 18 days. (k) The observation of starch granule structure in rice endosperm of WT, *lox10-1* mutants and *LOX10-OE1* overexpression lines with or without artificial aging for 18 days. Bar = 10 μm

Lipid peroxidation and degradation mediated by enzymes of the LOX pathway are thought to be important causes of seed aging. Therefore, we detected the expression levels of LOX pathway-related genes and the downstream metabolism-related genes related to seed vigor in the rice endosperm, and found that the expression levels of the LOX pathway-related genes *LOX1*, *LOX2* and *LOX3* and of the downstream metabolism-related genes *OsAOS2*, *OsHPL1* and *OsHPL3* were significantly higher in the two overexpression lines than in the WT (Fig. 5a-f). In plants, LOX usually catalyzes the oxidation of polyunsaturated fatty acids ( PUFAs ) and free fatty acids to produce unsaturated fatty acid hydroperoxides (Feussner and Wasternack 2002; Stelmach et al. 2001). Thus, we speculate that the free fatty acids concentration in the rice embryo may be associated with seed longevity during storage. To test this hypothesis, we used metabolomics to detect the concentration of free fatty acids in the embryos of WT, mutant *lox10-1* and overexpression line *LOX10-OE1* before and after aging. Interestingly, the concentrations of many different types of free fatty acids, including LNA and LA, were significantly lower in *lox10-1* than in *LOX10-OE1* and WT before as well as after aging (Fig. 5 g-j and S4). Furthermore, the concentrations of cis-11-Octadecenoic acid and stearic acid were much higher in *LOX10-OE1* seeds after artificial aging for 18 days (Fig.S4). These results suggest that when the expression level of LOX10 is inhibited, the hydroperoxide produced by LOX10 on linoleic acid and linoleic acid is also decreased, which affects the transport of calcium ions into the cells, and finally affects the activation of phospholipase A and the release of PUFAs from phospholipids. Overall, *LOX10* plays a critical role by influencing the LOX pathway and activating downstream metabolism-related genes, so that the disruption of *LOX10* increases seed longevity through reducing free fatty acid content in seed embryos before and after aging.

Previous studies had reported that down-regulation of *LOX3* expression affected grain starch granule structure (Xu et al. 2015). To investigate whether *LOX10* also affected starch granule morphology, we performed scanning electron microscopic (SEM) analysis on transverse sections of WT, *lox10-1*, *lox10-2*, *LOX10-OE1* and *LOX10-OE2* seed endosperms before and after artificial aging. Before artificial aging, the structure of the seed endosperms of WT, *lox10-1*, *lox10-2*, *LOX10-OE1* and *LOX10-OE2* was tightly arranged and the shape and size of cells were uniform and regular. After artificial aging for 18 days, the endosperm structure of the two *LOX10* overexpression lines and WT appeared to be loosely packed, fragile and irregular. In contrast, the endosperm structure of two *lox10* mutants after aging maintained a relatively complete and regular starch granule structure (Fig. 5k and S5). Therefore, the disruption of *LOX10* was closely associated with decreased stability of the morphological changes of rice endosperm starch granules in the presence or absence of artificial aging.

### Overexpression of *LOX10* increases tolerance of rice seedlings to saline-alkaline stress

**Fig. 6:**
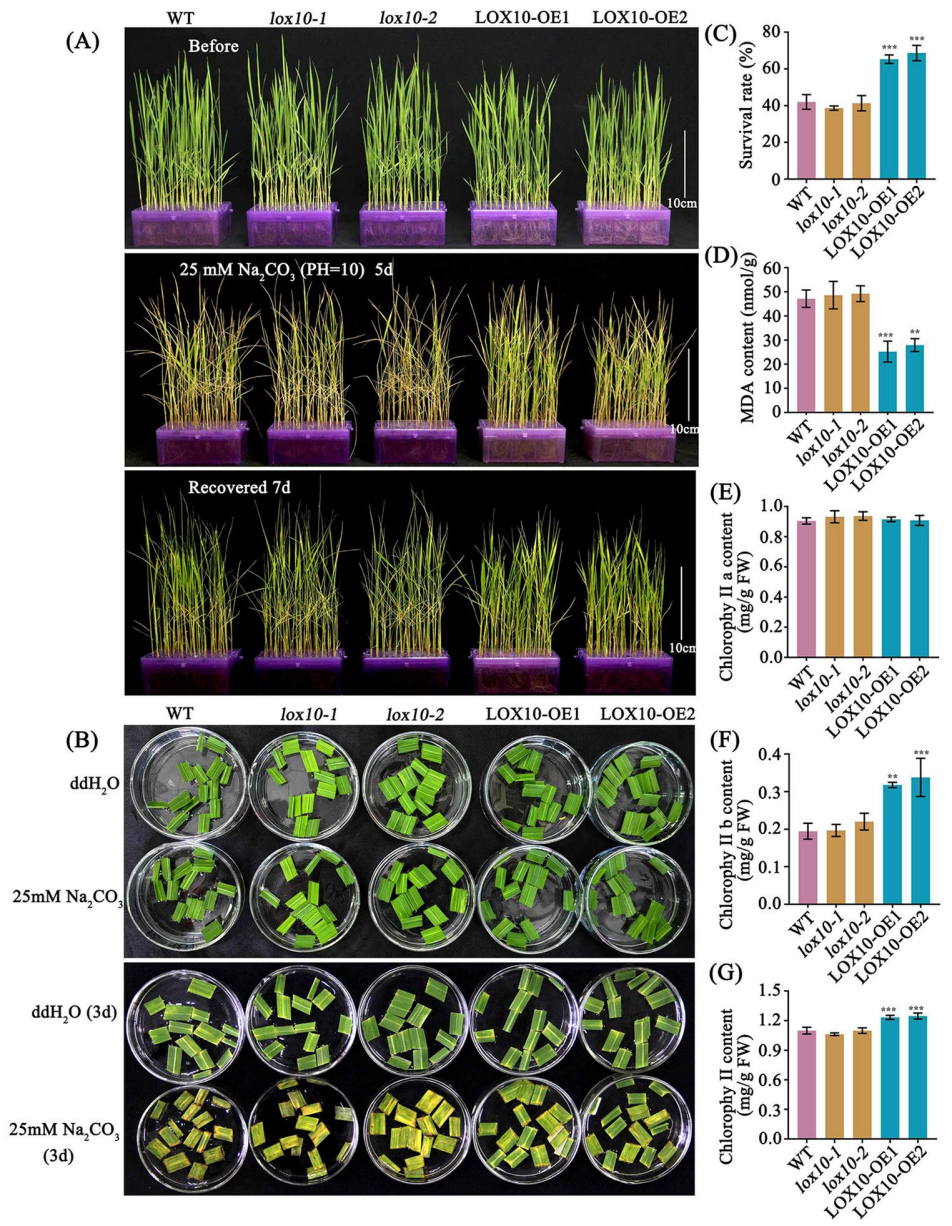
**Characterization of*LOX10*overexpression plants under saline-alkaline stress conditions** (a) Images of WT, *lox10* mutants and *LOX10* overexpression lines before and after recovery from the saline-alkaline treatment (25mM Na_2_CO_3_, pH = 10.0); (b) Images of flag leaf sections of WT, *lox10* mutants and *LOX10* overexpression lines before and after saline-alkaline treatment (25mM Na_2_CO_3_, pH = 10.0); (c) Survival rates; (d) Malondialdehyde (MDA) content of WT, *lox10* mutants and *LOX10* overexpression lines after 48 h of the saline-alkaline treatment (25 mM Na_2_CO_3_, pH = 10.0). (e-g) Chlorophyll a, chlorophyll b and total chlorophyll contents in WT, *lox10* knockout mutants and *LOX10* overexpression lines were determined after 48 h of the saline-alkaline treatment (25 mM Na_2_CO_3_, pH = 10.0). Values are means ± SD of three biological replicates; ** and *** indicate the significant difference compared to WT at 1% and 0.1% levels, respectively, according to Student’s t-test

Saline-alkaline soil is characterized by a combination of high sodium ion (Na^+^) concentration and high pH at the same time, which has more complex, adverse effects on plant growth and development than pH-neutral saline soils (Tang et al. 2014). It was found that LOX activity was higher and lipid peroxide oxidation was activated under salt stress conditions (Ben-Hayyim et al. 2001). As part of an investigation of the role of *LOX10* in saline-alkaline stress tolerance, the two *LOX10-OE* overexpression lines showed increased seedling survival rates under saline-alkaline conditions (25 mM Na_2_CO_3_, pH = 10.0), comparing with the two *lox10* mutants and WT lines (Fig. 6a, c). At the 25 mM Na_2_CO_3_ (pH = 10.0) concentration, the flag leaves of the mature plants of the *LOX10* overexpression lines remained green and with less necrosis or fewer spots than in WT or *lox10* mutant plants (Fig. 6b). To examine the concentration of malondialdehyde (MDA), a marker of oxidative stress-related lipid peroxidation, we performed MDA quantification after three-week-old rice seedlings had been treated with sodium carbonate for 48 h. The results showed lower concentrations of MDA in the two *LOX10-OE* overexpression lines than in the two *lox10* mutants and the WT lines (Fig. 6d). Meanwhile, we have measured and compared Chlorophyll a, chlorophyll b and total chlorophyll contents in WT, *lox10* knockout mutants and *LOX10* overexpression lines. And found that chlorophyll b and total chlorophyll contents of *LOX10* overexpression lines were significantly higher than WT and *lox10* knockout mutants (Fig. 6e-g). Taken together, these results indicate that the overexpression of *LOX10* plays an important role in determining rice tolerance to saline-alkaline stress.

## Discussion

Seed storability is an important agronomic and physiological trait in crop production. Seed storability has been shown to be regulated by genetic and environmental factors, but the mechanisms involved remain unclear. Previous studies had shown that temperature, moisture content and oxidation processes are the main factors affecting seed longevity (Ellis and Roberts 1980; Smith and Berjak 2017). In recent years, several studies have detected a number of quantitative trait loci (QTLs) related to seed storage longevity in rice, such as *qLG-2*, *qLG-4* and *qLG-9* (Miura et al. 2002). Artificial aging is widely used to study rice seed longevity by simulating natural conditions because of its simple operation, stable environment and ease of control (Priestley and Leopold 1983; Salama and Pearce 1993). In the current study, we cloned *LOX10* from rice ‘Kitaake’ and found that this gene played an major role in seed longevity and seedling salt-alkaline stress tolerance. Our study suggests that *LOX10* is located in the cytoplasm, and expression of the corresponding gene is induced to express during seed germination (Fig. 3j). Disruption of *LOX10* inhibits seed germination under normal conditions, on the contrary, after natural and artificial aging of seeds, the *lox10* knockout mutants maintained a higher germination potential than those of the WT and the overexpression lines (Fig. 2), without affecting other agronomic traits (Fig.S3a-f). These results are consistent with those from previously reported roles of *LOX2* and *LOX3* in the regulation of rice seed longevity (Huang et al. 2014; Xu et al. 2015). In addition, we also showed that *LOX10* overexpression lines enhanced the tolerance of rice seedlings to saline-alkaline stress (Fig. 6a-c).

In plants, the expression of the *LOX* genes is regulated by different factors, including phytohormones (Creelman and Mullet, 1997; Melan et al. 1993), biotic and abiotic stresses (Porta et al. 1999), and the plant species being studied. *LOX* gene show different, organ-specific expression patterns (Kolomiets et al. 2001). The hydroperoxy fatty acid products of LOX reaction can be further converted into different compounds through different enzyme pathways and thus have diverse functions (Feussner et al. 2001; Porta and Rocha-Sosa 2002). Our data showed that the expression levels of the LOX pathway-related genes *LOX1*, *LOX2* and *LOX3* and the downstream metabolism-related genes *AOS2*, *HPL1* and *HPL3* were significantly higher in the overexpression lines than in the WT and the *lox10* mutants (Fig. 5a-f). It is generally considered that LOXs in plant seeds act as a storage protein and do not have a clear physiological role (Siedow 1991). During germination, LOX proteins and their corresponding mRNAs accumulate and new LOXs are synthesized in seedlings and cotyledons (Melan et al. 1994; Porta et al. 1999). The storage lipids are mobilized from lipid bodies to release free fatty acids for further oxidation metabolism (Feussner et al. 2001). In vitro, most LOXs prefer free fatty acids as substrates (Feussner et al. 2001; Stelmach et al. 2001). By quantification of free fatty acid concentration in embryos before and after aging, we found that the free fatty acid concentrations of *lox10* mutant seeds embryos, including LNA and LA, were lower than in WT and the *LOX10* overexpression lines, through the detection of free fatty acid content in embryos before and after aging (Fig. 5 g-j). These results suggest that the concentration of free fatty acids in the embryo may be an important factor affecting seed storability. Seed germination is closely related to lipid degradation and hense LOX activity, but the underlying mechanism needs further study.

The LOX pathway of PUFAs plays an important role in regulating plant growth and development, and tolerance to biotic and abiotic stresses. The metabolites produced in the LOX pathway act as stress signaling molecules to perform diverse functions (Babenko et al. 2017; Viswanath et al. 2020), so that LOX activity can be used as a molecular marker for plant response to stress (Pokotylo et al. 2015; Porta and Rocha-Sosa 2002). The effects of cold and salt stress on 9-LOX and 13-LOX activity were different, suggesting that different abiotic stresses may regulate different LOX pathways (Babenko et al. 2017). It was reported that the LOX activity of salt-tolerant plants increased under salt stress conditions (Ben-Hayyim et al. 2001), whereas the expression of *OsLOX2* in rice was induced under drought stress (Du et al. 2013).

Our results showed that overexpression of *LOX10* increased the tolerance of rice seedlings to saline-alkaline stress, whereas the accumulation of MDA in the overexpression lines was also lower after 48 h exposure to sodium carbonate treatment compared with WT and the knockout mutants (Fig. 6d). At the same time, chlorophyll b and total chlorophyll contents of *LOX10* overexpression lines were significantly higher than WT and *lox10* knockout mutants (Fig. 6e-g). Thus, we confirmed that the overexpression of *LOX10* enhance the resistance of rice seedlings to alkaline stress. We also found that overexpression of *LOX10* enhance tolerance of rice seedlings to saline-alkaline, but the mutants showed no significant difference with WT, this may due to the decrease of *LOX10* expression in the two mutants was not as significant as the increase in the *LOX10* overexpression lines. Previous studies had reported that the concentration of free polyunsaturated fatty acids from LOX substrates increased in response to stress effects (Porta and Rocha-Sosa 2002). This indicates that the free fatty acid content of the LOX substrate has a positive effect on plant tolerance to stress.

In summary, our results provide new insights into the regulatory role of *LOX10* on seed longevity during storage and on the stress tolerance of rice seedlings. The knockout *lox10* mutants increase seed longevity by reducing the decomposition of free fatty acids during seed storage. However, the overexpression of *LOX10* increased the metabolism of free fatty acids, which produced different metabolites and activated various signaling molecules to enhance the tolerance of rice seedlings to saline-alkaline stress, but the functions of *LOX10* toward seed storability and stress response of rice seedlings warrant further investigation.

## Accession numbers

Sequence data used in this study are from the Rice Genome Annotation Project website (MSU, http://rice.uga.edu/index.shtml). The accession numbers are as follows: *LOX10*, LOC_Os11g36719; *LOX1*, LOC_Os03g49380; *LOX2*, LOC_Os03g5286; *LOX3*, LOC_Os03g49260; *OsAOS2*, LOC_Os03g12500; *OsHPL1*, LOC_Os02g12690; *OsHPL3*, LOC_Os02g02000.

## Electronic supplementary material

Below is the link to the electronic supplementary material.

Supplementary Material 1

## Data Availability

The datasets used and analyzed during the current study are available from the corresponding author on reasonable request.
