# Supplementary material for: The lipoxygenase OsLOX10 affects seed longevity and resistance to saline-alkaline stress during rice seedlings
Source: Plant Mol Biol. 2023 Mar 3;111(4-5):415–28. doi: 10.1007/s11103-023-01334-8 (PMC10089987; doi:10.1007/s11103-023-01334-8)
Supplement: Supplementary file 1 — Supplementary Material 1 [file 11103_2023_1334_MOESM1_ESM.docx]

**Supplementary material**


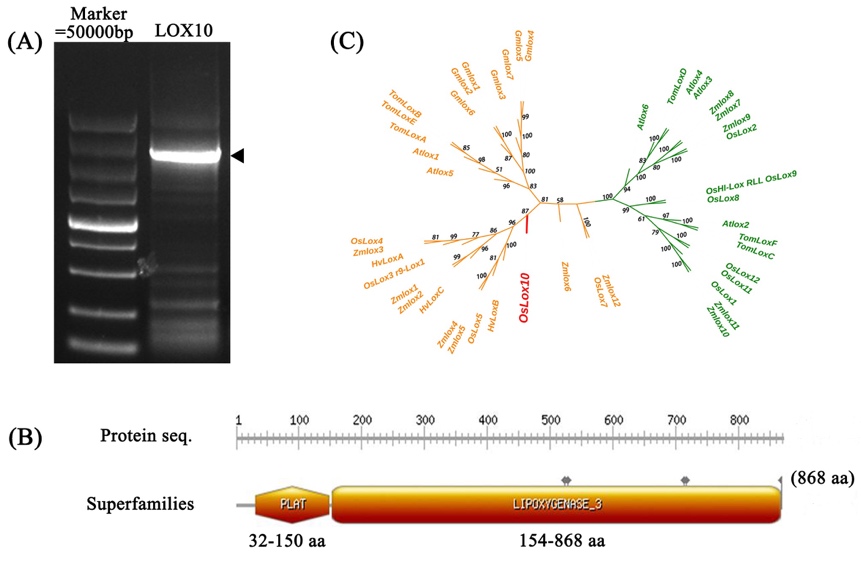


**Fig.S1 Characterization of *LOX10* in rice**

1. Cloning of intact *LOX10*; (b) The structure characterization and conserved domain analysis of LOX10 gene; (c) The phylogenetic tree analysis of *LOX10* gene; The Os: *Oryza sativa*, At: *Arabidopsis thaliana*, Zm: *Zea Mays*, Tom: Tomato, Gm: Soybean, Hv: Wheat.


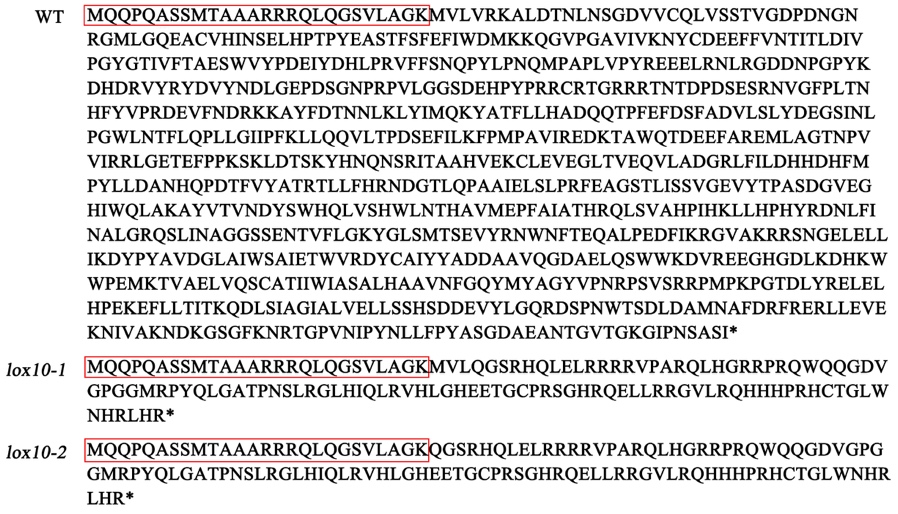


**Fig.S2 Comparison of amino acid sequences between WT and *lox10* mutants in rice.**

**
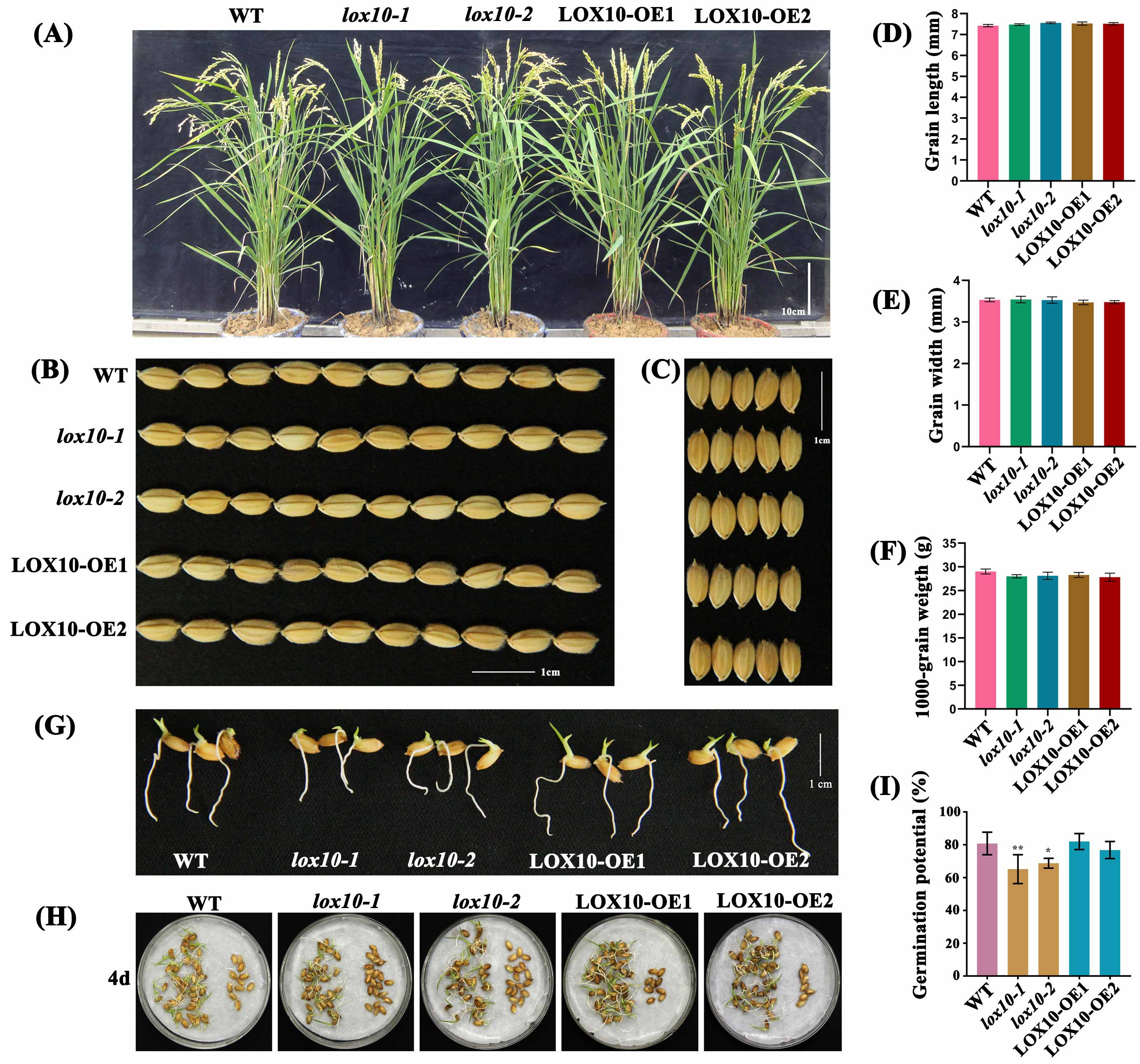
**

**Fig.S3 Comparison of agronomic traits between WT, *lox10* mutants and *LOX10* overexpression lines.**

1. Phenotypes of WT, *lox10* mutants and *LOX10* overexpression lines in the experimental field. Bar=10 cm. (b,c) Comparison of grain traits between WT, *lox10* mutants and *LOX10* overexpression lines. Bar=1 cm. (d) Grain length. (e) Grain width. (f) 1000-grain weight. (g) Seed germination of WT, *lox10* mutants and *LOX10* overexpression lines after 4 days. Bar=1 cm. (h, i) Germination potential of WT, *lox10* mutants and *LOX10* overexpression lines after 4 days.


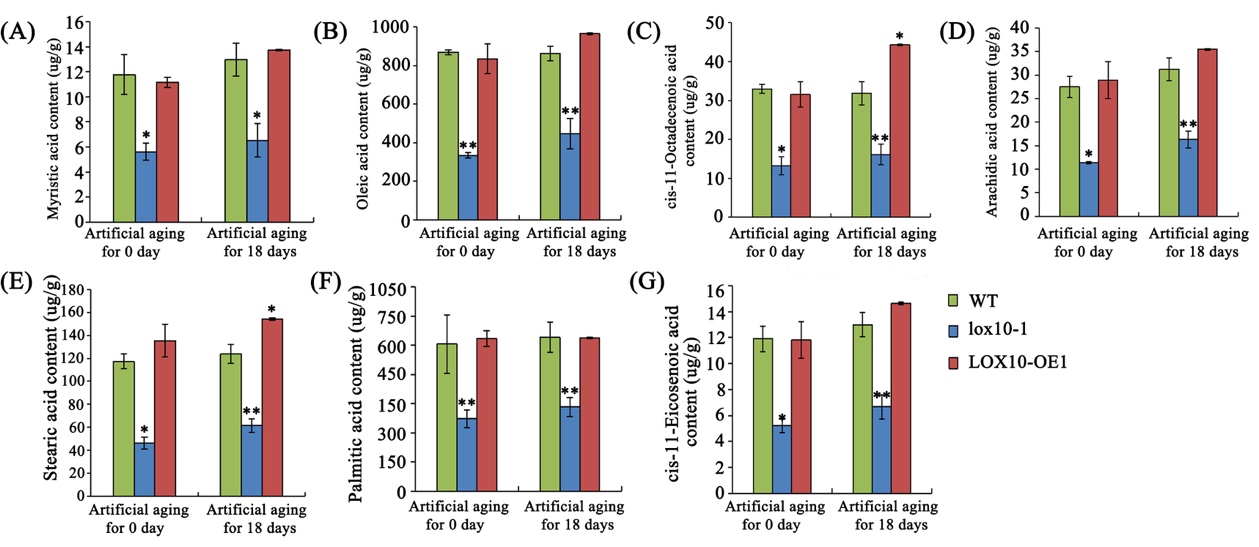


**Fig.S4 Determination of free fatty acid content in rice embryo of WT, *lox10* mutants and *LOX10* overexpression lines.**

1. Myristic acid content. (b) Oleic acid content. (c) cis-11-Octadecenoic acid content. (d) Arachidic acid content. (e) Stearic acid content. (f) Palmitic acid content. (g) cis-11-Eicosenoic acid content.


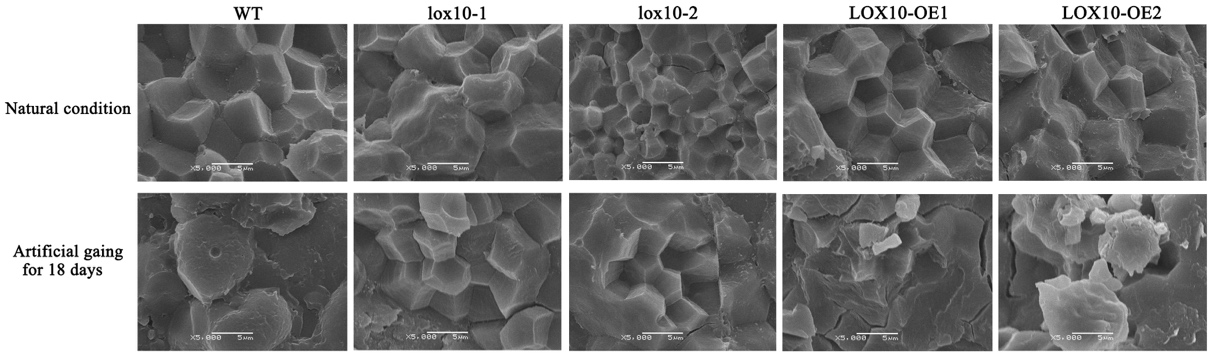


**Fig.S5 The observation of starch granule structure in rice endosperm of WT, *lox10-1* mutants and *LOX10-OE1* overexpression plant under natural condition and artificial aging for 18 days. Bar=5 um.**

**Table S1. Primers used in this study.**

| **Primer name** | **Sequence (5′ to 3′)** | **Function** |
| --- | --- | --- |
| OsU3-FD3 | GACAGGCGTCTTCTACTGGTGCTAC | Identification of transgenic *lox10* mutant plants |
| TaU3-RD | CTCACAAATTATCAGCACGCTAGTC |  |
| LOX10-OE-Hyg-F | CGAGAGCCTGACCTATTG | Identification of *LOX10* overexpression transgenic plants |
| LOX10-OE-Hyg-R | GCCTCCAGAAGAAGATGT |  |
| LOX10-CDS-F | AGGTAGCTAGTAGTACGATGCAG | Cloning of *LOX10* gene |
| LOX10-CDS-R | ACACGAGAAGAGAAATGTTGCT |  |
| LOX10-Pro-F | GACCAAGCTCCTGTTCCTCC | Cloning of *LOX10* gene promoter |
| LOX10-Pro-R | TTCAAGTTGGTGTCGAGAGC |  |
| LOX10-OE-NcoI-F | GGACTCTTGACCATGGCAGCAGCCGCAGGCGAG | Cloning pCAMBIA-1302, pairing with *LOX10*-OE-NcoI-F and *LOX10*-OE-BstEII-R |
| LOX10-OE-BstEII-R | GGAAATTCGAGCTGGTCACCTCAGATGGAGGCGCTG |  |
| LOX10-GFP-BamH-F | GATCCAGTGGGATCCATGCAGCAGCCGCAGGCG | Cloning pRTVcGFP , pairing with *LOX10*-GFP-BamH-F and *LOX10*-GFP-HindIII-R |
| LOX10-GFP-HindIII-R | CCGCACTAGTAAGCTTGATGGAGGCGCTGTTGGG |  |
| LOX10-GUS-BamH-F | GAGCTCGGTACCCGGCTCAGTTCGATCCCCTACC | Cloning pCAMBIA-1305, pairing with *LOX10*-GUS-BamH-F and *LOX10*-GUS-NcoI-R |
| LOX10-GUS-NcoI-R | TACCCTCAGATCTACCATGGTTCAAGTTGGTGTCGAGAGC |  |
| LOX10-28a-NdeI-F | GTGCCGCGCGGCAGCCATATGATGCAGCAGCCGCAGGCG | Cloning pET28a(+), pairing with *LOX10*-28a-NdeI-F and *LOX10*-28a-HindIII-R |
| LOX10-28a-HindIII-R | CTCGAGTGCGGCCGCAAGCTTTCAGATGGAGGCGCTGTTG |  |
| Actin-qPCR-F | AGTGTCTGGATTGGAGGAT | internal control |
| Actin-qPCR-R | TCTTGGCTTAGCATTCTTG |  |
| LOX10-qPCR-F | TTCCTTCTCACCATCACTAA | Relative expression level analysis of *LOX10* |
| LOX10-qPCR-R | GTTCTTCTCCACCTCCAA |  |
| LOX1-qPCR-F | GAGGAGATGGCACAGGTGTT | Relative expression level analysis of *LOX1* |
| LOX1-qPCR-R | TTGGATGCGGCTTCTCTACT |  |
| LOX2-qPCR-F | CTCAACGGACTCACAGTA | Relative expression level analysis of *LOX2* |
| LOX2-qPCR-R | GGCTGGAGGAAGACTAAG |  |
| LOX3-qPCR-F | AGAGCAAGGTCCATACAC | Relative expression level analysis of *LOX3* |
| LOX3-qPCR-R | GTCATTAACAGCAACATAAGC |  |
| OsAOS2- qPCR-F | CCTAGCGTTGACAACAAG | Relative expression level analysis of *OsAOS2* |
| OsAOS2- qPCR-R | TAAGCAGTACGATTGACG |  |
| OsHPL1- qPCR-F | AGTTCAACAAGCTCAACA | Relative expression level analysis of *OsHPL1* |
| OsHPL1- qPCR-R | GTGAAGCTGGAAGATGAG |  |
| OsHPL3-qPCR-F | GTACGTGTACTGGTCCAA | Relative expression level analysis of *OsHPL3* |
| OsHPL3- qPCR-R | CTTGTCGAGCTTGGTGAA |  |
